# Design of a Blockchain-Enabled Traceability System Framework for Food Supply Chains

**DOI:** 10.3390/foods11050744

**Published:** 2022-03-03

**Authors:** Lixing Wang, Yulin He, Zhenning Wu

**Affiliations:** 1 School of Computers and Engineering, Northeastern University, Shenyang 110000, China; hyl1026789105@163.com; 2 Laboratory for Soft Machines and Electronics, School of Packaging, Michigan State University, East Lansing, MI 48824, USA; 3 College of Information Science and Engineering, Northeastern University, Shenyang 110000, China; wuzhenning@ise.neu.edu.cn

**Keywords:** blockchain, RFID, food package, food supply chain

## Abstract

Tracing food products along the entire supply chain is important for achieving better management of food products. Traditionally, centralized traceability systems have been developed for such purposes. One major drawback of this approach is that different users of the supply chain have their own systems with their own complexities and distinct features; thus, the interaction among them creates challenges when implementing a single centralized system. Therefore, a decentralized traceability system is favorable for tracing food products along the supply chain. In this study, we develop a supply chain traceability system framework based on blockchain and radio frequency identification (RFID) technology. The system consists of a decentralized blockchain-enabled data storage platform for data management and an RFID system at the packaging level for data collection and storage. We applied a consortium blockchain to the application. Fabric 2.0 in Hyperledger was chosen as the development platform. The proposed blockchain-enabled platform can provide decentralized data management and its underlying algorithm can guarantee data security. The system includes a creatively designed blockchain-enabled data structure in the RFID tag. When people scan the tag, the relevant information is written in the tag as a block linked to the previous blocks; simultaneously, the information is transmitted to the blockchain platform and recorded on the platform. No battery is required and the system works when there is an RFID reader nearby. The usage conditions included shipment, stocking, and storage. The RFID tag can be directly attached to paper packaging. This approach embeds the blockchain technique into the RFID tag and develops a corresponding system. The new traceability system has the potential to simplify the tracking of products and can be scaled for industrial use.

## 1. Introduction

People often have contact with food products in their daily lives and food quality is a universal concern. Food quality problems also lead to serious social issues. Some examples of the most serious incidents include milk powder contaminated with melamine, pork contaminated with clenbuterol, Sudan dyes found in duck eggs, and recycled gutter oil used in cooking [1]. Most fresh food products are perishable. Their quality is sensitive to temperature and other environmental factors. Incorrect storage or transportation usually results in considerable economic loss to the corresponding companies. For example, 25–30% of food losses and damages are caused by inadequate transportation and distribution facilities such as cold storage units, dedicated fleets, and cold trucks [2]. In a report published by the United Nations, the National Development and Reform Commission [3] estimated that every year approximately one-third of all food items are lost globally with a value of approximately $8.3 billion. Consequently, tracing and tracking food products along the supply chain is an important topic for researchers.

The Internet of Things (IoT) technologies are immensely helpful in the food supply chain [4,5]. Radio frequency identification (RFID) is one of the most popular techniques. RFID has been increasingly used in logistics and supply chain management, because it can increase the efficiency of data input [6]. Between 2005 and 2006, an electronic pedigree (ePedigree) was proposed. It is an electronic document that provides data on the history of a particular product item. Therefore, RFID technology allows the tracking of items in real-time across the supply chain. This makes it easier to identify and trace products [7,8]. Jedermann, R. et al., developed a real-time autonomous sensor system for monitoring products when they were being transported [9]. Woo, S. H. et al., proposed an activity product state tracking system architecture for tracking products even when they were in a box or container [10].

With the widespread use of RFID, many researchers have designed systems to enhance food safety management. Abad, E. et al., developed an RFID-based system for the tracking and cold-chain monitoring of food [11]. The ePedigree was advocated to gain full supply chain visibility with detailed trace and track information. In another study, Wang, L. et al., proposed a system called the “supply-chain pedigree” [12].

Recently, the quick response (QR) code has also become a popular technology in traceability systems. Compared with barcodes, QR codes have a larger storage capacity without increasing costs. Compared with RFID, the cost of implementation of a QR code system is lower. People can easily obtain product information by scanning the code using a smartphone. There is no need to invest in dedicated reading devices such as RFID readers. For this application, Dong, Y. et al., applied QR codes to analyze China’s leafy vegetable supply chain [13]. Xu, Z. and Gao, H. M. used QR code technology to build a white guard traceability system for SHUNZI Vegetable Cooperatives in order to achieve farm-to-table whole process recordability and traceability [14]. Peng, Y. et al., presented a QR code based tracing method for a meat quality tracing system [15]. Li, Z. et al., combined RFID and QR code techniques together to realize real-time tracking and tracing for a prepackaged food supply chain [16].

Many approaches provide interactive and dynamic explorations for the public and governments to explore the information in each part of the food chain and provide easy implementation of hazard analysis and critical control points in the food industry. One major drawback of these approaches is the complexity and distinct features in the individual systems from different users/customers owing to the different requirements, facilities, and conditions among them, resulting in difficulty in adopting a centralized system for all customers. The emerging blockchain technique can help improve this situation. 

Blockchain was first proposed by Nakamoto, S. in 2008 [17]. The well-known cryptocurrency Bitcoin was created based on this technique. A blockchain is a distributed database. It comprises a series of orderly blocks that are chained together. Data are stored in blocks such as the ledger [18]. One defining characteristic of blockchain is decentralization. Many researchers have noticed this advantage and have applied it in supply chain management [19,20,21], including the food supply chain [22,23,24,25]. However, most studies have ignored data collection. Different companies still need to upload information to a centralized platform. Thus, a practical model incorporating decentralization has not been demonstrated.

In summary, it is necessary to trace and track food products along the supply chain. In recent years, several types of approaches to achieve this goal have been developed. However, each company has its own system, which makes it difficult to implement a traceability system for all the stakeholders along the supply chain. Any missing data in the links in the supply chain makes product tracing inaccurate. The integration of RFID and blockchain techniques in food packaging can help the implementation of a traceability system with different stakeholders; consequently, the objective of this study is to develop a packaging system framework for tracing and tracking food supply chains based on blockchain and RFID technologies. The system consists of a decentralized blockchain-enabled data storage platform for data management and an RFID system for data collection and storage. It works when there is an RFID reader nearby. The usage conditions include shipment, stocking, and storage. The new traceability system makes tracing and tracking products much easier and cheaper.

The remainder of this paper is organized as follows: Section 2 briefly reviews previous research on blockchain and RFID techniques in supply chains and food supply chains, in Section 3, the entire framework of the proposed system is introduced, Section 4 presents a case study to demonstrate the workflow of the proposed system, Section 5 presents the research conclusions and provides an outlook for future work.

## 2. Literature Review

**Figure 1:**
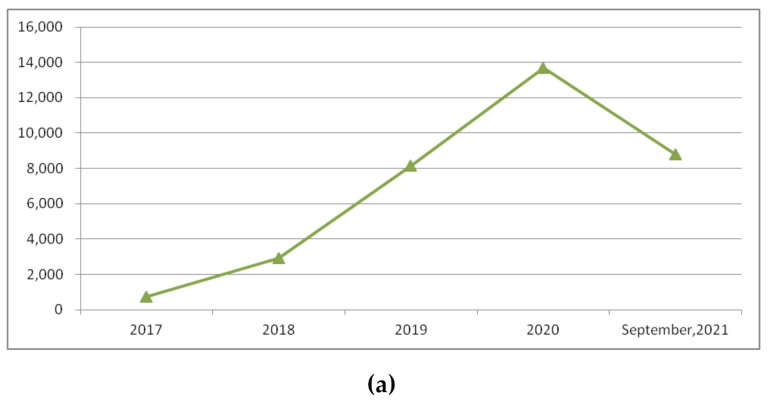

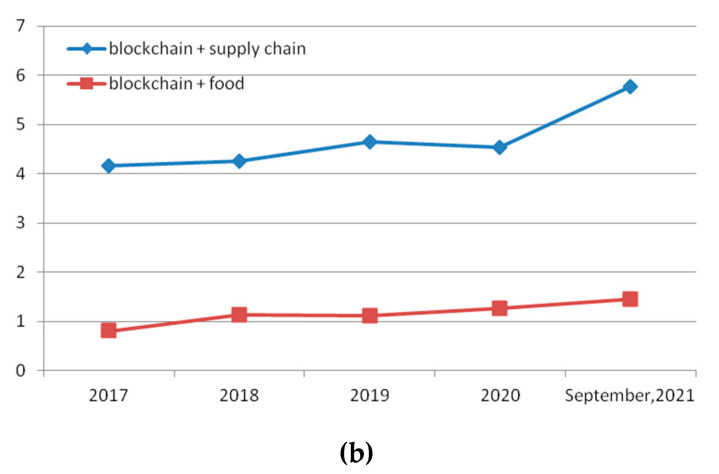
Publication trends: (**a**) blockchain, (**b**) percentages in publications about blockchain.

The extensive research interest in blockchain began in 2018. We looked at research records covering the last five years (2017–September 2021) using keyword searches such as “blockchain”, “blockchain + supply chain”, and “blockchain + food” to explore the Web of Science database. The results are listed in Table 1a. We also calculated the percentages of the publications with the latter two keywords in the publications with the keyword “blockchain”. The results are listed in Table 1b. The publication trends are shown in Figure 1. We can see that there is an increasing interest in blockchain research. Studies applying this technique to supply chains and food areas are increasing yearly. This means that researchers have noticed that this emerging technique has the potential to bring benefits to these areas. However, the limited number of publications suggests that the current studies remain in the early stages.

Decentralization makes blockchain technology attractive to researchers aiming to apply it to the supply chain, but there is little research to apply blockchain in supply chain management [26,27,28,29]. Smart contracts and consensus algorithms need to be redesigned for blockchain applications in supply chains [30,31,32]. Based on these studies, there are also some successful cases of blockchain applications in the supply chain. Wang et al., built a novel blockchain-based information management framework for a precast supply chain, thereby extending the applications of blockchain in the domain of construction supply chains [33]. Ho, G. T. et al., proposed a blockchain-based system for the accurate recording of aircraft spare parts traceability data with organizational consensus and validation using Hyperledger Fabric [34]. Omar, I. A. et al., presented a blockchain-based approach using Ethereum smart contracts and decentralized storage systems to transform vendor-managed inventory supply chain operations [35].

Insofar as studies targeting the food supply chain are concerned [36,37,38,39], among these studies how to apply blockchain for food traceability in the supply chain is a hot topic. Lin, Q. et al., designed a decentralized traceability system for food safety problems based on blockchain and an electronic product code information services network [40]. Tsang, Y. P. et al., proposed a blockchain–IoT based food traceability system that integrates a blockchain, IoT technology, and fuzzy logic for managing perishable food. A new consensus mechanism for considering the shipment transit time, stakeholder assessment, and shipment volume was developed to address the needs for food traceability, weight minimization, and vaporization characteristics [41]. Powell, W. et al., analyzed an ongoing beef supply chain project integrating the blockchain and IoT for supply chain event tracking and beef provenance assurance and proposed two solutions for increasing data integrity and trust in the blockchain and IoT-enabled food supply chain [42].

The aforementioned studies primarily focused on data management. However, data collection is also important for supply chain management. As stated in Section 1, RFID and QR codes are currently two popular techniques used in tracing and tracking systems to increase the efficiency of data input. Therefore, some studies have considered hybrid RFID approaches, QR codes, IOT techniques, and blockchains. Lakshmi, G. V. et al., used QR codes and a blockchain for inventory management [43]. Baralla, G. et al., developed a generic agri-food supply chain traceability system based on blockchain technology using Hyperledger Sawtooth. The system acquired data simply through a QR code scan [44]. Dey, S. et al., proposed a blockchain-technology-based framework, which made food product information easily accessible, traceable, and verifiable by consumers and producers by using QR codes to embed the information [45]. Bencic, F. M. et al., proposed a smart tag, called a DL-tag, which is used to track products during their lifecycle. The tags contain QR codes and the system implements a blockchain [46]. Compared with RFID, the cost of deploying a QR code system is much lower. However, the main drawback of QR codes is that they are easy to physically duplicate or destroy. There are safety risks in food supply chain management, where food safety is the main concern. In view of these different concerns, and in light of the current literature, we focused this research on a combination of blockchain and RFID technologies.

Feng, T. first utilized RFID and blockchain techniques to develop an agri-food supply chain traceability system framework. The system could realize traceability with trusted information across the entire agri-food supply chain, thereby effectively guaranteeing food safety by gathering, transferring, and sharing the relevant data in the production, processing, warehousing, distribution, and selling stages [47]. Meanwhile, the same author proposed the concept of “BigchainDB” [48], aiming to improve performance by applying blockchain for high-volume data storage. Helo, P. and Shamsuzzoha, H. M. considered the challenges of tracing and tracking in multi-company project environments. They proposed a pilot system formed by the combination of RFID, IoT, and blockchain technologies for real-time tracking and tracing of logistics and supply chains [49]. Mondal, S. et al., proposed a blockchain-inspired IoT architecture for creating a transparent food supply chain. The architecture was realized by integrating an RFID-based sensor at the physical layer and a blockchain at the cyber layer [50]. Mazzei, D. et al., described the implementation of a portable, platform-agnostic, secured blockchain tokenizer for industrial IoT trustless applications. The system was designed, implemented, and tested in two supply chain scenarios [51]. Sfa, A. et al., proposed two blockchain-based decentralized mutual authentication protocols for IoT systems. Moreover, combining blockchain with RFID can also help anti-counterfeiting efforts in the supply chain as these tags can be easily cloned in the post supply chain [52]. Toyoda, K. et al., proposed a novel product ownership management system for anti-counterfeiting RFID-attached products [53].

From the review, it can be found that most studies have considered the use of RFID for data collection, and blockchain for uploaded data processing and storage. In fact, these two techniques are generally used separately and only in the supply chain. Based on previous studies, this research focused on improving food packaging. We propose a traceability system framework for supply chain management. The system integrates blockchain with RFID much more deeply at the package level. The detailed design of the system is described in the following sections.

## 3. Traceability System Design

**Figure 2:**
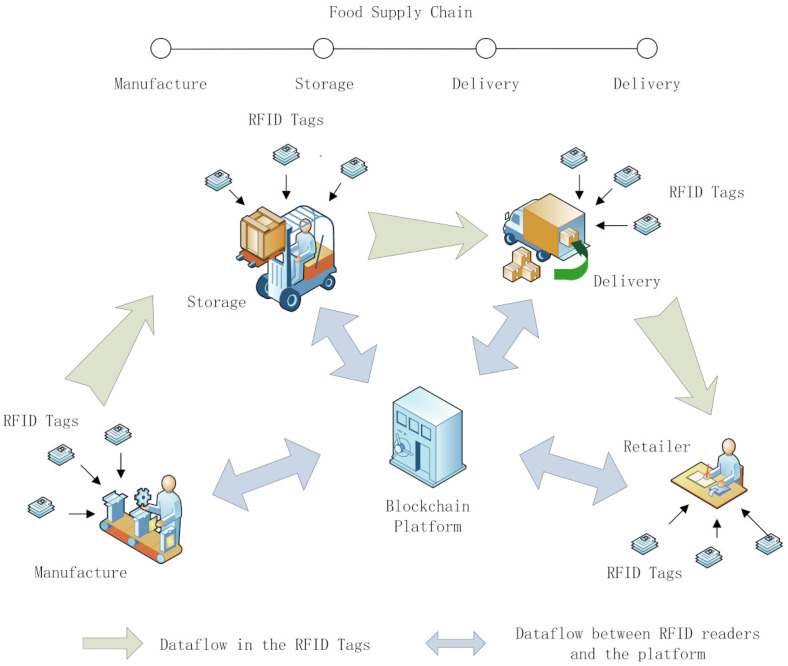
Application scenarios of the traceability system.

**Figure 3:**
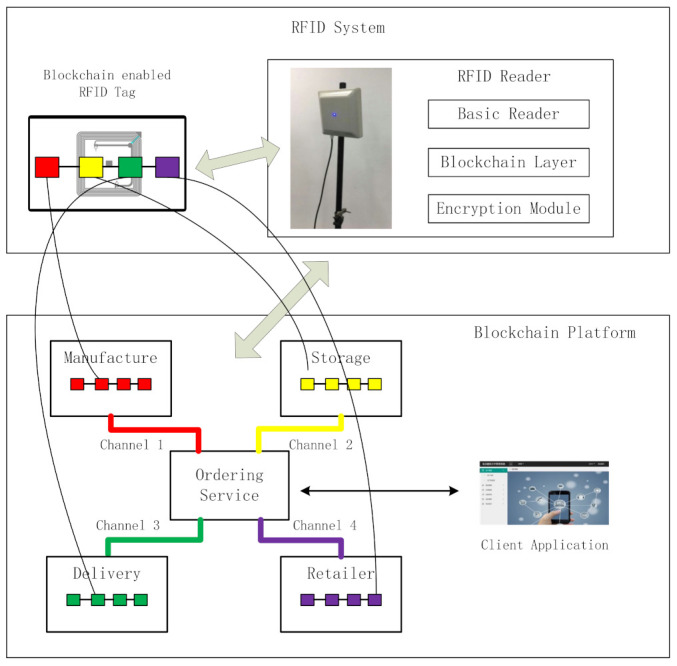
Traceability system architecture (The color is used to distinguish the blocks from different sessions in the supply chain.).

Figure 2 illustrates the application scenarios of the system. The system has three main parts. One part comprises blockchain-enabled RFID tags attached to the product packaging. The blockchain-enabled RFID tags follow the products along the entire supply chain. The relevant product information, transactions, storage, and delivery status are all recorded. Some tags can be coupled with sensors to measure and record environmental conditions. When the RFID tags are read by the RFID readers at each link of the supply chain, the information is transmitted to the blockchain light nodes. These comprise the second part of the system. The light nodes are integrated with the RFID readers. They are responsible for the communication and verification of RFID tags. Owing to the limited capacity of the RFID reader, the light node does not store all the ledgers. In addition, cross-validation is performed to guarantee safety. The approach is described in Section 3.3. The third part of the system comprises a blockchain platform. This platform stores all the ledgers. It also contains a designed consensus mechanism for making decisions on how to record ledgers. Users are also provided with an interface for product information inquiry. Figure 3 shows the relationship between these three parts, along with the system architecture.

### 3.1. System Architecture

As presented in Figure 3, the system comprises three parts: the RFID tags, RFID reader, and backend blockchain platform. A simple blockchain is contained in a blockchain-enabled RFID tag. Each time the tag passes a reader, a block is generated. The information written in the tag and the hash value of the previous block are calculated using the SHA-256 algorithm. The result is also written in the tag as the hash value of the current block. In this way, the blocks are individually linked. The second part is an RFID reader. Similar to the role of the normal RFID system, the RFID reader builds communication between the RFID tags and the backend system. In addition to the basic functions of a normal RFID reader, the reader in this blockchain-based traceability system contains a blockchain layer to justify whether the tag is a blockchain-enabled tag and for deciding what information should be written on the tag after encryption. An encryption module is also included in the RFID reader and is used to calculate the hash value of the input information. Finally, the RFID reader sends the information that the reader writes to the backend blockchain platform. The reader also stores parts of the ledgers of the blockchain platform as a light blockchain node. The third part is the blockchain platform. All the ledgers are stored on it. The main functions of the platform are data management and verification. A user application linked to the blockchain platform was also designed. Its main purpose is to provide a convenient user interface for inquiries regarding food product supply chain information.

### 3.2. Blockchain-Enabled Radio Frequency Identification (RFID) Tag Design

This is the base of the system. As stated in Section 2, in most previous studies integrating RFID and blockchain, RFID tags were only used to collect data. In this study, the RFID tags contain a blockchain data structure. The tags must be attached to all the products in the entire supply chain. When the reader reads a tag, the information is recorded and a block is formed. The blocks form a chain and are stored in the tags; consequently, people only need to scan the tags. Then, they can obtain all the information regarding the product and what it has experienced from source to destination. There is no need for a central database or system for management because all the information users might need is stored in the RFID tags. Moreover, the blockchain structure guarantees data safety. If anyone wanted to modify it, it would have to be modified from the beginning.

**Figure 4:**
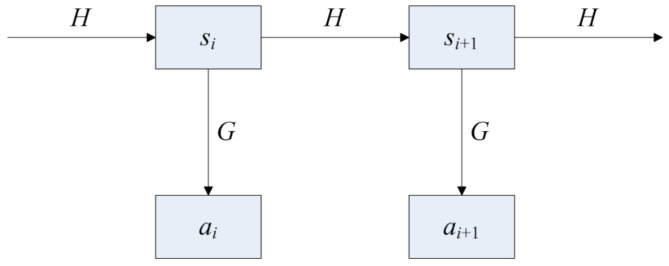
Safety mechanism of radio frequency identification (RFID).

With the explosion of chip technologies, the storage space in RFID tags has experienced exponential growth; consequently, it is possible for RFID tags to store all product information and transaction records, that is, much more than a mere ID number. Furthermore, the safety mechanism of an RFID system uses a hash chain scheme, as shown in Figure 4, which is the same as the basic principle of blockchain. In the hash chain, the tag and reader share two hash functions: *G*( ) and *H*( ). *G*( ) is used to calculate response messages. *H*( ) is used for updating. They also share an initial random identifier (*s*). When the RFID reader investigates the tag, the tag responds to the hash value $a_{i}=G\left(s_{i}\right)$ of the current identifier $s_{i}$; simultaneously, the tag updates the current identifier $s_{i}$ to $s_{i+1}=H\left(s_{i}\right)$. These two functions make it feasible to integrate blockchain with RFID.

**Figure 5:**
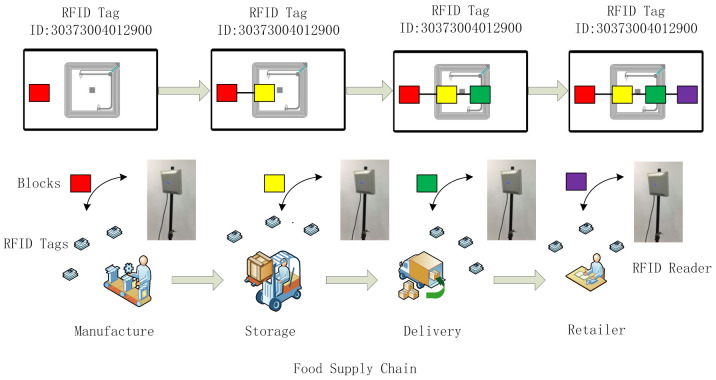
Work procedure of the RFID system (The color is used to distinguish the blocks from different sessions in the supply chain.).

The blockchain design in the RFID tags and the corresponding mechanisms are discussed below. When a food product is packaged, all the relevant information is coded by the hash algorithm and then recorded in the block; this block is called the “genesis block”. The block is stored in an RFID tag attached to the food package. When the product enters the next session, for example, storage in the warehouse, the relevant information is coded by the hash algorithm (SHA-256) and then recorded in a second block linked to the genesis block. When the product proceeds to the next session, the same work is repeated until the product reaches the consumer. Figure 5 illustrates the procedure. The data structure design of the blocks is presented in Table 2. It contains an RFID tag ID, reader IP address, product information, and relevant sensor data. The table also provides an example of the data (the only example). In fact, there is no need to fill all the columns or follow the data format. In addition to the necessary reader IP address, location, timestamp, and hash value the reader in each link of the supply chain only needs to input the information it can provide. In this way, it effectively solves the data-sharing problem of different companies with different types of information systems in a complex supply chain and at a low cost. The only thing the manufacturer needs to do is update the RFID reader at the software level.

### 3.3. Blockchain Light Node Design

The RFID reader takes the role of a bridge between the RFID tag and the blockchain platform. The main function of this system is communication. For the RFID tag, the RFID reader is responsible for reading the data of the tags, decrypting the data, and recording it in the database of the corresponding system. This system is also responsible for writing data in the RFID tags and for encryption. Meanwhile, ledgers in a period relevant to when the tags were read are also stored in the reader. If there are any data safety problems, the reader can also provide cross-verification. For a given (limited) storage capacity, the ledgers stored in the reader have a maximum size requirement. An RFID reader is a blockchain light node. Therefore, we need to develop a blockchain platform with full functionality. For the blockchain platform, the RFID reader includes a communication module for data processing and uploading.

For the communication with RFID tags, the work procedure is as follows:

Step 1: The RFID reader reads the RFID tag and determines whether it is a blockchain-enabled tag. If it is, proceed to Step 2; otherwise, the normal RFID reader procedure is completed.

Step 2: The reader determines whether the tag has been read before. If it has, proceed to Step 3. If it has not, proceed to Step 5.

Step 3: The reader determines whether the time interval between reads is longer than a user-defined value, t. If it is, proceed to Step 4. If it is not, proceed to Step 5.

Step 4: The reader reads the hash value from the second to the last block, then proceeds to Step 6.

Step 5: The reader reads the hash value of the last block.

Step 6: The reader writes the data into a tag with the hash value of the last step.

Step 7: The reader calculates the hash value of the input information using the SHA-256 algorithm.

Step 8: The reader writes the hash value into the tag.

**Figure 6:**
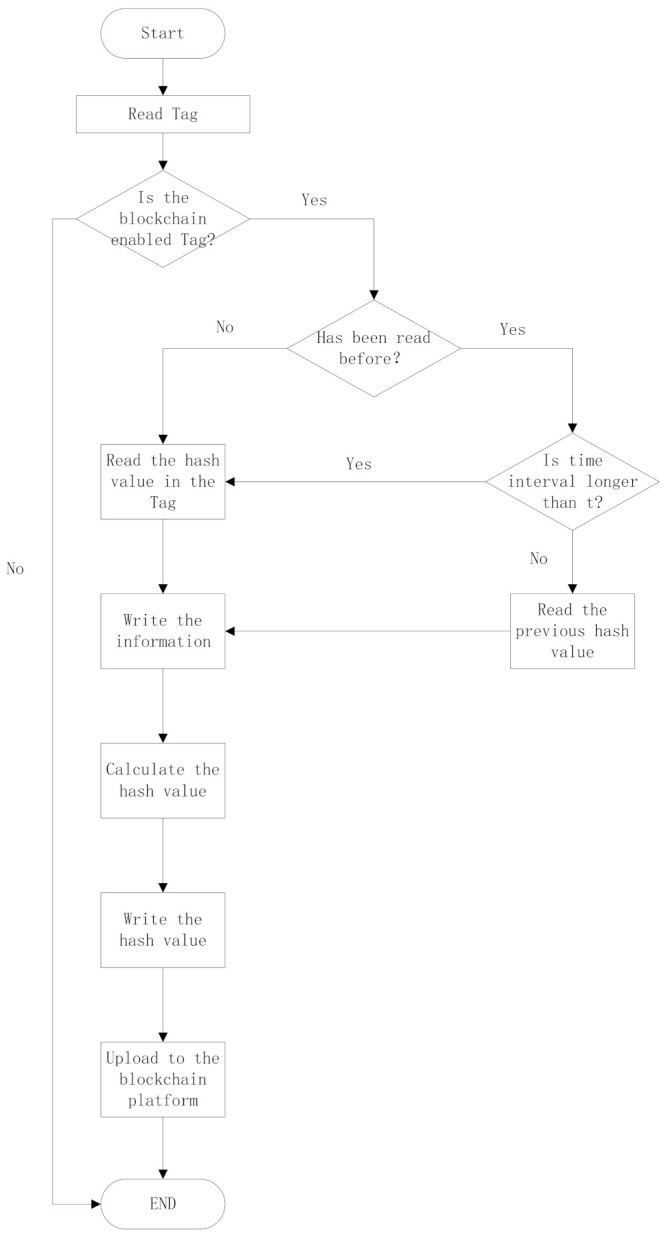
Workflow of the RFID reader.

Step 9: After data processing, the reader transforms the data into a blockchain platform. The communication module is described in the next section. Figure 6 shows a flowchart of how the RFID reader writes the data in the RFID tags.

**Figure 7:**
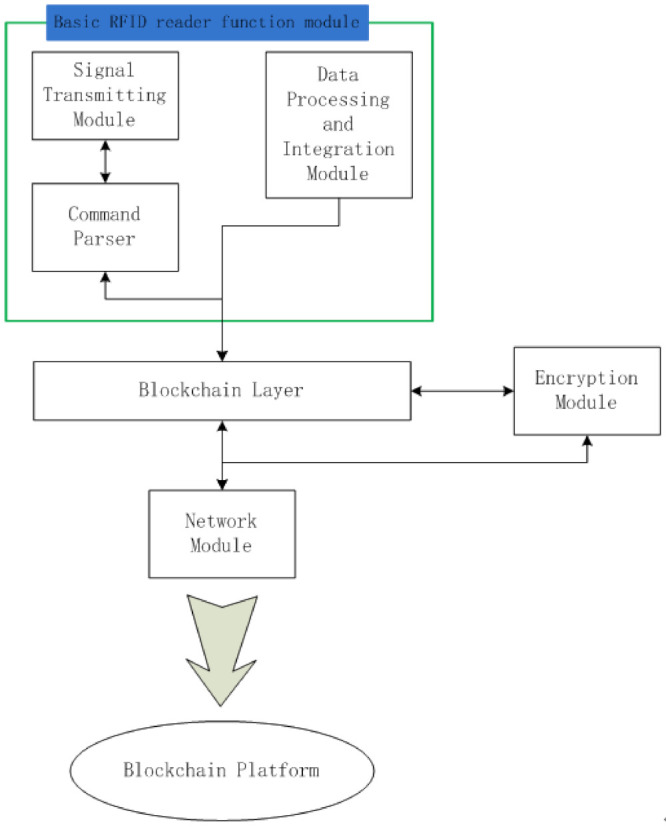
Data operational process to the blockchain platform.

The communication module in the RFID reader comprises four parts. The first part includes a radio signal transmitting and processing module, command parser, and data integration and processing module, which realize the basic RFID reader function. If the reader finds that the tag it has read is a blockchain-enabled RFID tag, the data are transmitted to the blockchain layer in the RFID reader. This is the second part of the communication module and represents its core. The blockchain layer reads the hash value of the last block in the tag and sends it, together with the new information it wants to write to the tag, to the third part of the communication module: the encryption module. The encryption module uses the SHA-256 algorithm to calculate a new hash value derived from the previous hash value and the input data. After encryption, this new (now current) hash value and input data are transmitted back from the blockchain layer, via the RFID reader, to the RFID tag as a new block. The blockchain layer also stores recent ledgers relevant to the tags it has read (within the limits of its storage capacity). When there is any verification requirement, these recorded ledgers can be used for cross-validation. The fourth part is the network module. It connects the reader to the Internet. Then, the hash value, input data, tag ID, and product ID can be transmitted to the blockchain platform. The recent ledgers of this RFID system will also be downloaded and updated from the blockchain platform. Figure 7 shows the architecture of the communication module between the light node and blockchain platform. This module also solves the problems of data integration, processing, and bulk data upload.

### 3.4. Blockchain Platform Design

**Figure 8:**
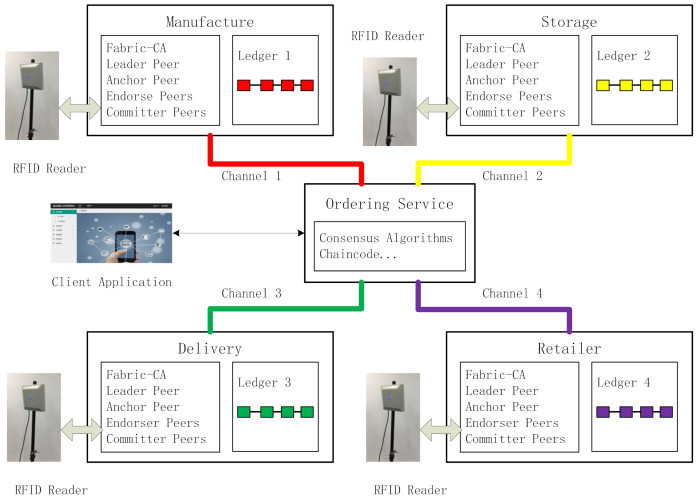
System architecture (The color is used to distinguish the blocks from different sessions in the supply chain.).

A blockchain platform was developed on Hyperledger Fabric. The system architecture is illustrated in Figure 8. The central part of the architecture is an ordering service. It organizes different parties in the supply chain actions for verification. Consensus algorithms and chain codes are both located here. The ordering service node also connects to a client application, as introduced in Section 3.5.

According to the characteristics of the supply chain, the parties can be divided into four groups: manufacturers, warehouses, logistics, and retailers. Each group is the responsibility of a different company. Channels are built between the group and ordering service; consequently, there are four channels in this platform, thus there are four ledgers. Each group contains the fabric certification authority, leader peer, anchor peer, endorser peers, and committer peers. The endorser peers and committer peers are connected to the responding RFID readers. RFID readers are the light nodes of their peers, as they only store parts of the ledgers. All the peer nodes, regardless of the company they belong to, store the entire ledger for their group. The records can also be the reader data, transmitted back by the RFID reader, and set as blocks linked together by a hash value. They are saved in their respective ledgers. In contrast to the client application, the tag ID in the ledger is set as the inquiry number. In fact, the RFID tag in the platform can also be considered as a peer node, however it contains parts of all four ledgers along with their relevant smart contracts.

**Figure 9:**
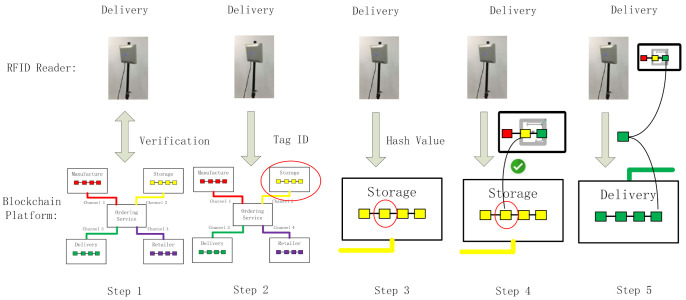
Workflow of the communication between the RFID reader and blockchain platform (The color is used to distinguish the blocks from different sessions in the supply chain).

The workflow for the data transmitted from the RFID reader to the platform is illustrated in Figure 9. First, the platform verifies the validity of the reader. Second, the RFID tag ID is extracted for the platform to identify the block of the RFID tag from the previous session. Then, the platform compares the hash value recorded in the last block of this RFID tag with the hash value of the previous block in the tag transmitted back by the RFID reader. If they are identical, all the information transmitted back is recorded as a block of that tag and linked with other blocks in the same group. If they are different, a warning is issued.

### 3.5. Client Application Design

We also designed a client application for the traceability system (the platform was developed in Chinese; we translated the words in the following figures for clarity). This application only provides an interface for users for inquiries. There is no need for companies in the product supply chain to use it; they only need to write relevant information on the tag through the RFID reader.

**Figure 10:**
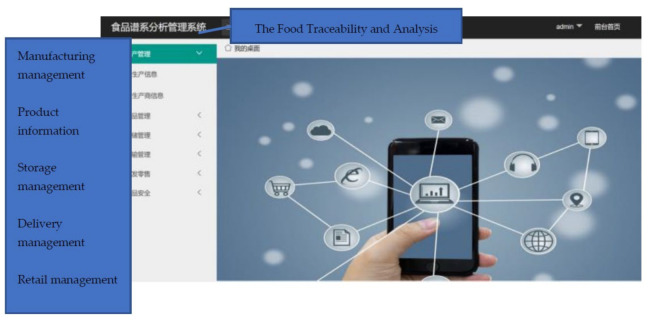
Client Application Interface.

As shown in Figure 10, there are six modules in the application: product information, manufacturing management, storage management, delivery management, retail management, and food safety analysis. The product information module includes basic product information such as shelf life and name. The manufacturing management module processes the information from the manufacturer. The details can be decided by the company itself, for example, details about storage management, delivery management, and retail management. The relevant parties in the supply chain can only provide their company names or more detailed information such as the storage environment, delivery truck, and arrival time. The final part is the food safety analysis module. In this module, the food pedigree is analyzed to locate the key node in the supply chain. If a product has any problems, the module can also help to recall the problem product and analyze which chain has the problem. Detailed information can be found in our previous study [12,54].

In this user application, all modules contained the product ID. The product ID is the index number for this system. The tag ID and hash value are not shown to the users owing to safety considerations.

## 4. Case Study

In this section, a case study is presented to demonstrate the operation of the system. The case is based on Tiandi Yuan Ltd., a famous beverage company. Their main products are white wines.

**Figure 11:**
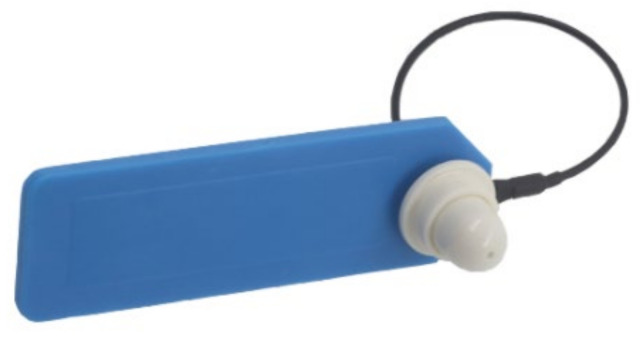
The attached UHF RFID Tag.

**Figure 12:**
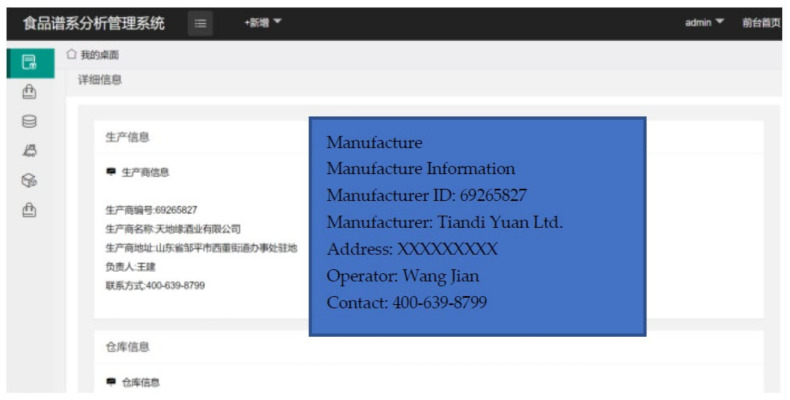
Detailed Information in Client Application.

When a product is manufactured, a serial number is generated. We use the example “6926582705239LW-2013-0300001” for demonstration. Furthermore, a UHF RFID tag, as shown in Figure 11, is also attached to the product package. An RFID reader scans the tag and the tag ID is linked to the product ID. The system uploads relevant product information to the system, as shown in Figure 12. A hash value is calculated using SHA-256 with the input of the tag ID and the product information. The hash value is written on the tag by the RFID reader as the genesis block (block 0). The RFID reader also connects to the blockchain platform and transmits the tag ID, product information, and hash value to the platform. These inputs and the previous hash value of the block in the platform are calculated using SHA-256. Then, a new block is generated in the platform. Parts of the ledger of the manufacturing node are also downloaded onto the RFID reader. When the product enters the next session, the same steps are repeated. In the storage session, the reader reads the tag, links the tag ID with the storage information, and uploads the information to the system. Meanwhile, the reader uses the information and the hash value recorded in block 0 to generate a new hash value associated with block 1 to be recorded in the RFID tag. The rest of the procedure is the same as in the manufacturing session. The procedure for the remaining sessions is also the same for each session.

**Figure 13:**
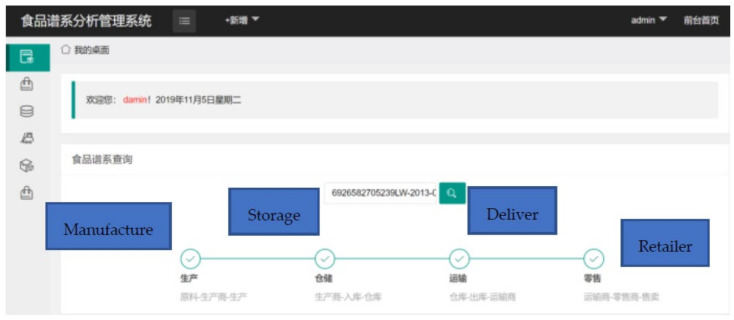
Food Supply Chain in Client Application.

Finally, as shown in Figure 13, when the consumer inputs the product ID at the interface, the application shows the entire supply chain of the product. The user can also find detailed information by clicking the nodes in the supply chain or clicking the buttons in the left column.

This company has different partners in its product supply chain, especially in logistics and retailer sessions. In the past, it was difficult for companies to follow their products along the entire supply chain, because not all companies are willing to upload the information to the company’s information platform. With the help of this system, the company has better information about its products in the supply chain, such as final consumers. The company merely needs to update its partners’ RFID readers. The reader can automatically identify the blockchain-enabled RFID tag in order to perform its functions, as stated above. Although some companies in the supply chain are resistant to sharing information, basic information such as time and location will also be recorded automatically through the RFID reader. With a more transparent supply chain, the company can better manage its products. The data also helps the company analyze its product supply chain. If there are any problem products, they can be easily recalled. Moreover, the application of blockchain techniques in both the platform and the RFID tags significantly increases product security and facilitates anti-counterfeiting measures.

The case study proves that the proposed system can effectively help different companies with different information system collect and upload data to the blockchain plat-form. With the information, all the traces of a product along the supply chain can be clearly presented to the consumers. Furthermore, the system also has the potential to enable companies to manage their products better with some data analysis modules.

## 5. Conclusions

This research proposed a blockchain and RFID-enabled traceability system for the food industry. The system consists of three parts. The first is a blockchain–RFID tag. We deeply integrated blockchain and RFID techniques. A simple blockchain storage structure was designed for data storage in an RFID tag based on the original safety scheme of the RFID system. All information regarding the product as it follows the supply chain is recorded in the tags in a blockchain format. The second part is an RFID reader with a blockchain light node. Its main function is to build communication between the RFID and backend blockchain platforms in a normal RFID system. In addition to these basic functions, the reader also contains blockchain and encryption modules. These two modules change the reader to a blockchain light node. The blockchain layer can determine whether a tag is a blockchain-enabled RFID tag. If it is, the data will be dealt with by the encryption module and written on the tag with a calculated hash value. Moreover, the recent ledgers of the tags that the RFID reader has read previously are also stored in it. If there is any uncertainty about the data, the reader can also help in cross-validation. The third part is the blockchain platform. The platform was developed using Hyperledger Fabric. It stores all the ledgers. The reader transmits the data written on the tags to the platform. The platform manages and verifies the data and the reader. Finally, we designed a user application which has the look and feel of a webpage. It provides a user interface for convenient inquiries regarding the pedigrees of food products. Detailed information on each chain, as provided by the responding company, can also be obtained using this application.

The main contribution of this research is the creative design of a blockchain-based RFID tag. The tag can be attached to a food package with low costs for the application of this new emerging blockchain technique. Blockchain technology allows supply chain monitoring to be decentralized. For the various companies referred to in the complex supply chain, there is no need to use different types of information systems or to follow different types of data formats for different companies. They need to (and can simply) update their RFID readers at the software level. Anyone requiring the product supply chain information can download it from the blockchain platform. With the help of blockchain, the food supply chain can be more transparent and can be easily traced and tracked.

Comparisons between our proposed system and other popular blockchain-based methods for tracing and tracking systems are shown in Table 3. From the comparison, it can be seen that our proposed system has a high security level and relatively low cost, allowing convenient data input, and it needs to be equipped with RFID readers without simultaneously demanding a uniform information system.

We did not redesign the consensus mechanism for blockchain platforms. The system is only in the research phase, therefore there is no problem with the current volume of data. However, a traditional consensus mechanism, such as practical Byzantine fault tolerance, may not satisfy the data throughput in the supply chain. The data in the supply chain have their own characteristics. In future studies, we will consider developing a new consensus mechanism that is more suitable for the food supply chain. We will also consider printing QR codes on the RFID tags. Companies without RFID readers can also be involved in this system. Moreover, with the help of the blockchain technique, the food supply chain becomes much more transparent and easier to safeguard. The analysis of these data will also be our research focus in the next phase.

## Figures and Tables

**Table 1:** (**a**) Absolute numbers of recent publications regarding blockchain and relevant applications, (**b**) percentages of recent publications about blockchain computing related to food or supply chains (based on keyword search).

**(a)**					
Keywords	2017	2018	2019	2020	January–September 2021
Blockchain	743	2933	8144	13,685	8811
Blockchain + Supply Chain	31	125	379	620	508
Blockchain + Food	6	33	91	173	128
**(b)**					
Keywords	2017	2018	2019	2020	January–September 2021
Blockchain + Supply Chain	4.17%	4.26%	4.65%	4.53%	5.77%
Blockchain + Food	0.81%	1.13%	1.12%	1.26%	1.45%

**Table 2:** Sample record for a single product data structure design.

**Data Structure**	**Manufacture**	**Storage**	**Delivery**	**Retailer**
Tag ID	30373004012900	30373004012900	30373004012900	30373004012900
Reader IP	175.160.137.100	127.155.155.100	165.129.69.105	168.122.53.107
Timestamp	2110231409	2110231611	2110250632	2110261017
Product Name	Milk	Milk	Milk	Milk
Product ID	30373004740000	30373004740000	30373004740000	30373004740000
Location	39.773, 119.253	38.476, 117.245	36.279, 118.366	34.773, 113.727
Company	A	B	C	D
Job	Manufacture	Storage	Delivery	Retailer
Sensor	Not Applicable	Temperature	Temperature	Temperature
Sensor Data	Not Applicable	5	7	3
Previous Hash	Not Applicable	3a6fed5fc11392b3ee9f81caf017b48640d7458766a8eb0382899a605b41f2b9	d807aa9812835b01243185be550c7dc372be5d7480deb1fe9bdc06a7c19bf174	748f82ee78a5636f84c878148cc7020890befffaa4506cebbef9a3f7c67178f2
Hash Value	3a6fed5fc11392b3ee9f81caf017b48640d7458766a8eb0382899a605b41f2b9	d807aa9812835b01243185be550c7dc372be5d7480deb1fe9bdc06a7c19bf174	748f82ee78a5636f84c878148cc7020890befffaa4506cebbef9a3f7c67178f2	

**Table 3:** Comparisons between different blockchain based methods for tracing and tracking systems.

Approaches	Security Level	Convenient for Data Input	Cost
Blockchain	High	No (data need to be input manually)	Low
Blockchain + QR code	Low	Yes (But data need to be scanned individually)	Low
Blockchain + RFID	Medium	Yes (Each stakeholder needs to connect their RFID system to a uniform information system)	Medium
Blockchain + IoT	High	Yes (Each stakeholder should guarantee the device can be connected to the Internet)	High
Our method	High	Yes (Each stakeholder merely needs an RFID reader)	Medium
